# Adherence to the 2017 Clinical Practice Guidelines for Pediatric Hypertension in Safety-Net Clinics

**DOI:** 10.1001/jamanetworkopen.2023.7043

**Published:** 2023-04-14

**Authors:** Allison J. Carroll, Yacob G. Tedla, Roxane Padilla, Arjit Jain, Eduardo Segovia, Anoosh Moin, Andrea S. Wallace, Olutobi A. Sanuade, Craig B. Langman, Nivedita Mohanty, Justin D. Smith

**Affiliations:** 1 Department of Psychiatry and Behavioral Sciences, Northwestern University Feinberg School of Medicine, Chicago, Illinois; 2 Vanderbilt University School of Medicine, Nashville, Tennessee; 3 AllianceChicago, Chicago, Illinois; 4 Ann & Robert H. Lurie Children’s Hospital of Chicago, Chicago, Illinois; 5 Division of Health Systems and Community-Based Care, University of Utah College of Nursing, Salt Lake City; 6 Department of Population Health Sciences, Division of Health System Innovation and Research, Spencer Fox Eccles School of Medicine at the University of Utah, Salt Lake City; 7 Northwestern University Feinberg School of Medicine, Chicago, Illinois

## Abstract

**Importance:**

The 2017 Clinical Practice Guideline (CPG) for the diagnosis and management of pediatric hypertension (PHTN) categorizes a greater proportion of children with elevated blood pressure and PHTN, yet several barriers to CPG adherence have been noted.

**Objective:**

To assess adherence to the 2017 CPG for the diagnosis and management of PHTN and use of a clinical decision support (CDS) tool to calculate blood pressure percentiles.

**Design, Setting, and Participants:**

This cross-sectional study used electronic health record–extracted data from January 1, 2018, to December 31, 2019, among patients visiting 1 of 74 federally qualified health centers in AllianceChicago, a national Health Center Controlled Network. Children and adolescents (aged 3-17 years; hereinafter referred to as children) who attended at least 1 visit and had at least 1 blood pressure reading at or above the 90th percentile or diagnosis of elevated blood pressure or PHTN were eligible for data to be included in the analysis. Data were analyzed from September 1, 2020, to February 21, 2023.

**Exposures:**

Blood pressure at or above the 90th or 95th percentile.

**Main Outcomes and Measures:**

Diagnosis of PHTN (*International Statistical Classification of Diseases and Related Health Problems, Tenth Revision* [*ICD-10*], code I10) or elevated blood pressure (*ICD-10* code R03.0) and CDS tool use; blood pressure management (antihypertensive medication, lifestyle counseling, referral); and follow-up visit attendance. Descriptive statistics described the sample and rates of guideline adherence. Logistic regression analyses identified patient- and clinic-level associations with guideline adherence.

**Results:**

The sample consisted of 23 334 children (54.9% boys; 58.6% White race; median age, 8 [IQR, 4-12] years). Guideline-adherent diagnosis was observed in 8810 children (37.8%) with blood pressure at or above the 90th percentile and 146 of 2542 (5.7%) with blood pressure at or above the 95th percentile at 3 or more visits. The CDS tool was used to calculate blood pressure percentiles in 10 524 cases (45.1%) and was associated with significantly greater odds of PHTN diagnosis (odds ratio, 2.14 [95% CI, 1.10-4.15]). Among 15 422 children with blood pressure at or above the 95th percentile, antihypertensive medication was prescribed to 831 (5.4%), lifestyle counseling was provided to 14 841 (96.2%), and blood pressure–related referrals were given to 848 (5.5%). Guideline-adherent follow-up was observed in 8651 of 19 049 children (45.4%) with blood pressure at or above the 90th percentile and 2598 of 15 164 (17.1%) with blood pressure at or above the 95th percentile. Differences in guideline adherence by patient- and clinic-level factors were observed.

**Conclusions and Relevance:**

In this study, fewer than 50% of children with elevated blood pressure had a guideline-adherent diagnosis code or attended guideline-adherent follow-up. Using a CDS tool was associated with guideline-adherent diagnosis, but the tool was underused. Further work is needed to understand how to best support implementation of tools promoting PHTN diagnosis, management, and follow-up.

## Key Points

**Question:**

To what extent are clinicians adhering to the 2017 Clinical Practice Guideline and using a clinical decision support tool to calculate blood pressure percentiles for pediatric hypertension diagnosis, management, and follow-up?

**Findings:**

In this cross-sectional study of 23 334 children and adolescents (aged 3-17 years) with elevated blood pressure, less than half had a corresponding diagnosis in their medical record or attended the recommended follow-up visit. Using the clinical decision support tool was associated with higher rates of diagnosis, but the tool was underused.

**Meaning:**

These findings suggest that pediatric hypertension and elevated blood pressure remain underdiagnosed and undertreated among children and adolescents, which may have negative health consequences in adulthood.

## Teaser

This cross-sectional study examines the rates of adherence to the 2017 Clinical Practice Guideline for the diagnosis, management, and follow-up of hypertension and use of a clinical decision support tool to calculate blood pressure percentiles in children and adolescents.

## Introduction

The prevalence of pediatric hypertension (PHTN) is estimated to be approximately 4% globally^1^ and has risen in recent years,^2^ likely due to multiple factors (eg, high rates of overweight and obesity).^2,3^ This rise is due in part to the American Academy of Pediatrics 2017 Clinical Practice Guideline (CPG) for the diagnosis and management of PHTN,^4,5^ which replaced the 2004 Fourth Report.^6^ According to an analysis of patients aged 8 to 17 years, 1.5% to 2.5% more children and adolescents met criteria for PHTN per the 2017 CPG vs the Fourth Report.^7^ Compared with the Fourth Report, diagnosis adhering to the 2017 CPG significantly improved estimation of adult hypertension from 13% to 22%.^8^

Research shows that children with elevated blood pressure and PHTN frequently go undiagnosed.^9,10,11^ One study^12^ found that only 14.6% of children who met diagnostic criteria (per the Fourth Report) for elevated blood pressure or PHTN had a corresponding *International Statistical Classification of Diseases and Related Health Problems, Tenth Revision* (*ICD-10*), diagnosis code in the electronic health record (EHR). Another EHR study^10^ found that although blood pressure was nearly always measured at well-child visits, less than half of children with elevated blood pressure received appropriate, guideline-adherent management. Accurate diagnosis is critical for appropriate treatment planning to reduce the risk of future health consequences, as elevated blood pressure in children is associated with impairments in cardiac (eg, increased left ventricular mass) and vascular (eg, increased arterial stiffness) structures,^8,13,14^ as well as neurocognitive and psychological effects.^15^ Consequently, children with PHTN are at greater risk of developing hypertension, metabolic syndrome, and left ventricular hypertrophy in adulthood.^16,17^

Numerous barriers to PHTN diagnosis and management have been identified, including high patient volumes and short visit times in primary care; low confidence in blood pressure readings due to poor measurement technique or inadequate equipment; and lack of patient follow-up.^18,19^ In medicine broadly, heuristic decision-making and implicit bias among clinicians can affect treatment recommendations, particularly among patients who are members of historical minority groups.^20,21^ Clinical decision support (CDS) tools in the EHR have been shown to save time and decrease clinician decision-making burden, leading to improved recognition of PHTN.^22,23^

To date, most studies examining PHTN guideline adherence, as well as use of CDS tools, have been conducted using the Fourth Report. Our primary aim was to examine the rates of adherence to the 2017 CPG for PHTN diagnosis, management (antihypertensive medication, lifestyle counseling, referral), and follow-up. Our secondary aim was to assess associations with guideline adherence, including patient and clinic characteristics and the use of a CDS tool in the EHR designed to facilitate guideline adherence and patient and clinic characteristics.

## Methods

This report follows the Strengthening the Reporting of Observational Studies in Epidemiology (STROBE) reporting guideline for observational studies. All procedures were approved by Northwestern University’s Institutional Review Board, which waived the need for informed consent owing to the use of retrospective data.

### Setting

This study extracted data from the EHR of AllianceChicago, a Health Center Controlled Network of 74 federally qualified health centers across 18 states (eFigure 1 in Supplement 1) in urban, suburban, and rural settings with high proportions of Black and Hispanic or Latino populations. Patients seen at federally qualified health centers within the AllianceChicago network come from primarily uninsured, underinsured, and low-income populations.^24^

### Study Period

Data from visits that occurred between January 1, 2018, and December 31, 2019, were retrieved. This time frame was chosen because the recommendations from the 2017 CPG for hypertension were implemented through health information technology tools throughout the AllianceChicago network, including an EHR-based CDS tool, starting January 1, 2018.

### Participants

This study included 23 334 children and adolescents (aged 3-17 years; hereinafter described as children) who attended at least 1 health care visit in the AllianceChicago network during the study period and had at least 1 systolic or diastolic blood pressure reading at or above the 90th percentile (aged <13 years) or systolic blood pressure of at least 120 mm Hg and diastolic blood pressure of at least 80 mm Hg (aged ≥13 years) during the study period, and/or had an *ICD-10* diagnosis for elevated blood pressure (code R03.0: elevated blood pressure reading, without diagnosis of hypertension) or PHTN (code I10: essential [primary] hypertension). Patients were excluded if they were pregnant or diagnosed with heart disease.

### Data Collection

Participants in the AllianceChicago network use a centrally hosted, common EHR system (AthenaPractice). All visit information, patient characteristics, vital signs, and orders are recorded in the EHR.

#### Patient and Clinic Characteristics

The following patient characteristics were extracted as coded in the EHR: sex (boys or girls), age (3-6, 7-11, or 12-17 years), race (Asian, Black, Hispanic or Latino [where included as a race code in the EHR], White, multiracial, other [where included as a race code in the EHR], or unknown), ethnicity (Hispanic or Latino, non-Hispanic or non-Latino, or unknown), and weight status per body mass index (BMI) percentile (<5% [underweight], ≥5% to <85% [normal weight], ≥85% to <95% [overweight], or ≥95% [obesity]). Clinic characteristics included the setting (urban, suburban, or rural).

#### CDS Tool Use

The CDS tool, available starting January 1, 2018, was designed to automate calculation of blood pressure percentiles, classify blood pressure values, identify abnormal values, provide guidance on obtaining manual blood pressure values, and calculate mean values per the 2017 CPG (further information is provided in the eMethods and eFigure 2 in Supplement 1). Use of the CDS tool was ascertained per observations terms generated when clinicians clicked the Blood Pressure Interpretation Tool. The CDS tool was coded 1 if the observation term indicated use at all visits with an available blood pressure value and coded 0 if not used at 1 or more visits with an available blood pressure value.

#### Guideline-Adherent Diagnosis

A guideline-adherent diagnosis was coded if:

1. The child had at least 1 systolic or diastolic blood pressure value at or above the 90th percentile if younger than 13 years, systolic blood pressure at least 120 mm Hg or diastolic blood pressure at least 80 mm Hg if 13 years or older (hereafter referred to as blood pressure ≥90th percentile), and an elevated blood pressure diagnosis (*ICD-10* code R03.0); or
2. The child had at least 3 systolic or diastolic blood pressure values at or above the 95th percentile if younger than 13 years, systolic blood pressure at least 130 mm Hg or diastolic blood pressure at least 80 mm Hg if 13 years or older (hereafter referred to as blood pressure ≥95th percentile), and a PHTN diagnosis (*ICD-10* code I10).

#### Guideline-Adherent Management

The management options for elevated blood pressure included (1) antihypertensive medications, (2) lifestyle counseling, and (3) referrals to specialists. We extracted data concerning antihypertensive medication prescription (eg, angiotensin-converting enzyme inhibitors, β-blockers) (a complete list is provided in the eMethods in Supplement 1) at the time of the visit. We extracted data concerning receipt of lifestyle counseling (*ICD-10* code Z71.3: dietary counseling and surveillance; *ICD-10* code Z71.82: exercise counseling; or *Current Procedural Terminology* code 99401, 99402, 99403, or 99404: preventative medicine counseling and/or risk factor reduction intervention[s] provided to an individual). We extracted data concerning receipt of a hypertension-relevant referral (eg, cardiology, nephrology).

#### Guideline-Adherent Follow-up Visits

The 2017 CPG recommends that patients with blood pressure at or above the 90th or 95th percentile attend a follow-up visit within 6 months or within 2 weeks, respectively. Thus, patients with blood pressure at or above the 90th and 95th percentiles were included if they attended a first visit between January 1, 2018, and June 30, 2019, and were coded as attending guideline-adherent follow-up if they had a second visit within 6 months of the first. Patients with blood pressure at or above the 95th percentile were included if they attended a first visit between January 1, 2018, and November 30, 2019, and were coded as attending guideline-adherent follow-up if they had a second visit within 1 month of the first (to conservatively allow for 2-week follow-up, consistent with prior studies^25^).

### Statistical Analysis

Data were analyzed from September 1, 2020, to February 21, 2023. Descriptives were calculated for patients’ baseline characteristics. For the primary aim, rates of guideline-adherent diagnosis, management, and follow-up visit attendance per the 2017 CPG were calculated. Outcomes for guideline-adherent diagnosis included (1) elevated blood pressure diagnosis among children with at least 1 blood pressure measurement at or above the 90th percentile and (2) PHTN diagnosis among children with at least 3 blood pressure measurements at or above the 95th percentile. Outcomes for guideline-adherent management included (1) antihypertensive medications, (2) lifestyle counseling, and (3) referrals for children with blood pressure measures at or above the 90th percentile as well as children with blood pressure measures at or above the 95th percentile. Outcomes for guideline-adherent follow-up included 6-month follow-up attendance among children with blood pressure measures at or above the 90th percentile and 1-month follow-up attendance among children with blood pressure measures at or above the 95th percentile. For the secondary aim, multivariable logistic regressions were used to identify associations with guideline-adherent diagnosis, management, and follow-up. Covariates included in all models were patient sex (boys or girls), age (in years), race (Asian, Black, Hispanic or Latino, White, multiracial, other, or unknown race), ethnicity (Hispanic or Latino, non-Hispanic or non-Latino, or unknown ethnicity), BMI status (underweight, normal weight, overweight, or obesity), clinic setting (urban, suburban, or rural), and CDS tool use (yes or no; diagnosis models only). All analyses were conducted using Stata, version 17 (StataCorp LLC).^26^ A 2-tailed *P* < .05 was considered statistically significant.

## Results

### Sample Description

The sample, consisting of 23 334 children with blood pressure at or above the 90th percentile on at least 1 visit, included 12 811 boys (54.9%) and 10 523 girls (45.1%) with a median age of 8 (IQR, 4-12) years, and of diverse racial (715 [3.1%] Asian, 6084 [26.1%] Black, 1417 [6.1%] Hispanic or Latino [where included as a race code in the EHR], 13 663 [58.6%] White, 316 [1.4%] multiracial, and 284 [1.2%] other [where included as a race code in the EHR]) and ethnic (11 978 [51.3%] Hispanic or Latino and 10 687 [45.8%] non-Hispanic or non-Latino) backgrounds (Table 1). Of those, 15 422 children (66.1%) had blood pressure measured at or above the 95th percentile on at least 1 visit and 2542 (10.9%) had blood pressure measured at or above the 95th percentile on at least 3 visits.

**Table 1.** Sample Characteristics of Children and Adolescents Aged 3 to 17 Years With at Least 1 BP Reading at or Above the 90th Percentile Between January 1, 2018, and December 31, 2019

Characteristic	Patient data (N = 23 334)^a^
Age, y	
Median (IQR)	8 (4-12)
≤6	8139 (34.9)
7-11	9356 (40.1)
≥12	5839 (25.0)
Sex	
Boys	12 811 (54.9)
Girls	10 523 (45.1)
Race	
Asian	715 (3.1)
Black	6084 (26.1)
Hispanic or Latino^b^	1417 (6.1)
White	13 663 (58.6)
Multiracial	316 (1.4)
Other^b^	284 (1.2)
Unknown	855 (3.7)
Ethnicity	
Hispanic or Latino	11 978 (51.3)
Non-Hispanic or non-Latino	10 687 (45.8)
Unknown	669 (2.9)
BMI status	
Underweight	486 (2.1)
Normal	9727 (41.7)
Overweight	3800 (16.3)
Obesity	9321 (39.9)
Clinic setting	
Urban	16 863 (72.3)
Suburban	3794 (16.3)
Rural	2677 (11.5)

Abbreviations: BMI, body mass index; BP, blood pressure.

^a^ Unless otherwise indicated, data are expressed as No. (%) of children and adolescents. Percentages have been rounded and may not total 100.

^b^ Entered as a race code in the electronic health record.

### Guideline-Adherent Diagnosis

Guideline-adherent diagnosis results are presented in Table 2 and the Figure, A. Of the full sample, only 8810 children (37.8%) had a corresponding *ICD-10* diagnostic code (R03.0). Compared with children without the diagnosis code, those with the diagnosis code were more likely to be older (4534 of 5839 children [77.7%] aged ≥12 years vs 1893 of 8139 children [23.3%] aged ≤6 years); Black (2479 of 6084 [40.7%]) vs White (5314 of 13 663 [38.9%]) race and White vs Hispanic or Latino (442 of 1417 [31.2%]), other (53 of 284 [18.7%]), or unknown (243 of 855 [28.4%]) race; Hispanic or Latino (5054 of 11 978 [42.2%]) or unknown (239 of 669 [35.7%]) vs non-Hispanic or non-Latino (3517 of 10 687 [32.9%]) ethnicity; classified with obesity (4337 of 9321 [46.5%]) vs normal (2948 of 9727 [30.3%]) BMI; and have attended a clinic in an urban (7208 of 16 863 [42.7%]) vs suburban (1177 of 3794 [31.0%]) or rural (425 of 2677 [15.9%]) setting. There was no significant difference in diagnosis by sex.

**Table 2.** Logistic Regression Associations With Having a Guideline-Adherent *International Statistical Classification of Diseases and Related Health Problems, Tenth Revision *Diagnosis Code for Elevated BP or PHTN in the Electronic Health Record Among Children With Elevated BP Readings

Variable	Diagnosis of elevated BP in children with BP ≥90th percentile on ≥1 visit^a^			Diagnosis of PHTN in children with BP ≥95th percentile on ≥3 visits^b^		
	No. (%)	AOR (95% CI)	*P* value^c^	No. (%)	AOR (95% CI)	*P* value^c^
Age, y						
≤6	1893 (23.3)	1.24 (1.23-1.25)	<.001	7 (1.4)	1.16 (1.08-1.24)	<.001
7-11	2383 (25.5)			20 (2.0)		
≥12	4534 (77.7)			119 (11.4)		
Sex						
Boy	5043 (39.4)	1 [Reference]		91 (6.0)	1 [Reference]	
Girl	3767 (35.8)	1.00 (0.94-1.06)	.97	55 (5.4)	0.73 (0.51-1.05)	.09
Race						
Asian	212 (29.7)	1.10 (0.90-1.35)	.34	7 (8.3)	2.44 (0.94-6.43)	.07
Black	2479 (40.7)	1.26 (1.13-1.39)	<.001	44 (6.8)	1.73 (0.98-3.05)	.06
Hispanic or Latino	442 (31.2)	0.45 (0.39-0.51)	<.001	7 (7.9)	1.56 (0.67-3.64)	.30
White	5314 (38.9)	1 [Reference]		79 (5.1)	1 [Reference]	
Multiracial	67 (21.2)	0.78 (0.57-1.07)	.13	4 (10.8)	4.43 (1.34-14.61)	.02
Other	53 (18.7)	0.57 (0.40-1.80)	.001	2 (5.6)	1.39 (0.30-6.43)	.68
Unknown	243 (28.4)	0.78 (0.64-0.96)	.02	3 (3.5)	1.32 (0.36-4.85)	.68
Ethnicity						
Hispanic or Latino	5054 (42.2)	1.70 (1.54-1.88)	<.001	65 (5.5)	1.10 (0.65-1.89)	.72
Non-Hispanic or non-Latino	3517 (32.9)	1 [Reference]		79 (6.1)	1 [Reference]	
Unknown	239 (35.7)	1.24 (1.02-1.52)	.03	2 (3.5)	0.60 (0.14-2.67)	.50
BMI status						
Underweight	108 (22.2)	0.83 (0.65-1.06)	.14	1 (2.5)	1.36 (0.17-10.72)	.77
Normal	2948 (30.3)	1 [Reference]		24 (3.2)	1 [Reference]	
Overweight	1417 (37.3)	1.09 (0.99-1.19)	.08	18 (5.4)	1.43 (0.75-2.73)	.28
Obesity	4337 (46.5)	1.25 (1.17-1.34)	<.001	103 (7.3)	1.55 (0.96-2.49)	.07
Clinic setting						
Urban	7208 (42.7)	1 [Reference]		95 (5.5)	1 [Reference]	
Suburban	1177 (31.0)	0.55 (0.50-0.60)	<.001	29 (7.4)	1.27 (0.79-2.05)	.32
Rural	425 (15.9)	0.25 (0.22-0.29)	<.001	22 (6.4)	1.87 (1.02-3.40)	.04
CDS tool						
Not used	4077 (27.4)	1 [Reference]		28 (1.9)	1 [Reference]	
Used	4733 (56.1)	1.49 (1.38-1.59)	<.001	118 (10.8)	2.14 (1.10-4.15)	<.001

Abbreviations: AOR, adjusted odds ratio; BMI, body mass index; BP, blood pressure; CDS, clinical decision support; PHTN, pediatric hypertension.

^a^ Of 23 334 eligible children and adolescents, 8810 (37.8%) had a diagnosis of elevated BP in the electronic health record.

^b^ Of 2542 eligible children and adolescents, 146 (5.7%) had a diagnosis of PHTN in the electronic health record.

^c^ Compared with the reference category, values indicate the association with having a guideline-adherent *International Statistical Classification of Diseases and Related Health Problems, Tenth Revision *diagnosis code documented in the electronic health record.

**Figure.**
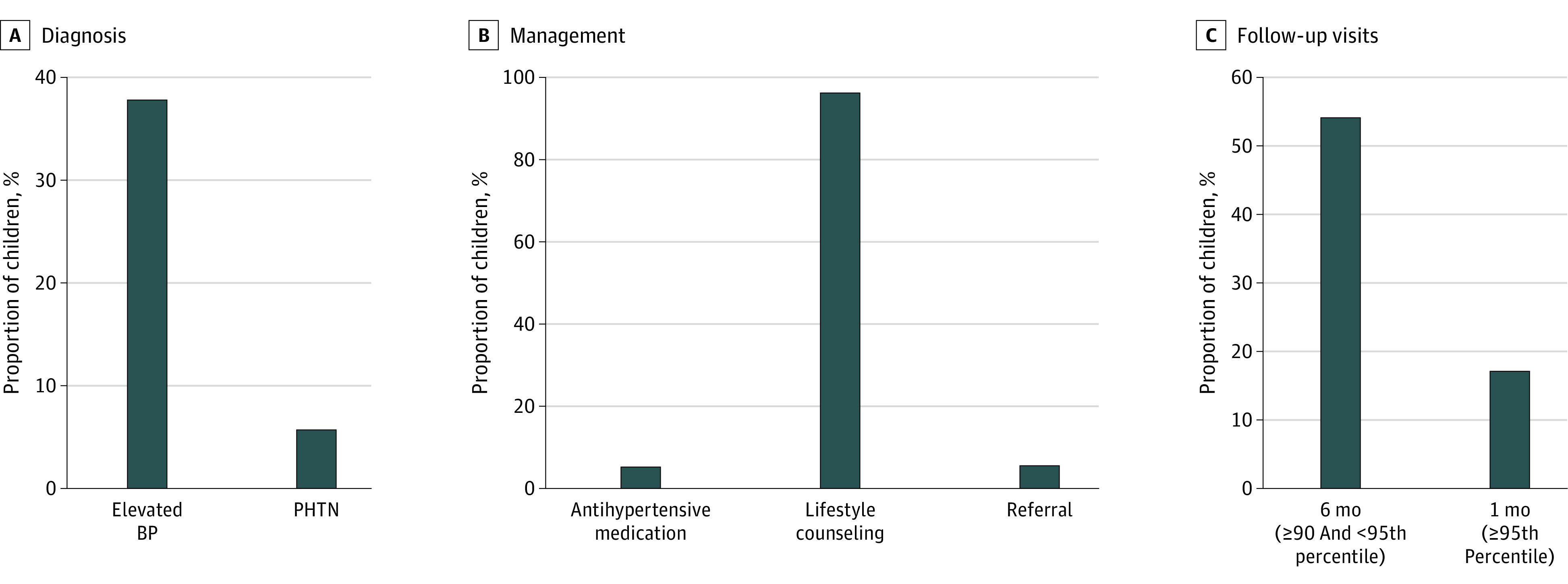
Proportions of Children With Guideline-Adherent Diagnosis, Management, and Follow-up Documented in the Electronic Health Record Elevated blood pressure (BP) was defined as at least 1 BP measurement at or above the 90th percentile; pediatric hypertension (PHTN), at least 3 BP measurements at or above the 95th percentile.

Of 11 675 children who attended at least 3 visits during the study period, 2542 (21.8%) had blood pressure measurements at or above the 95th percentile at 3 visits or more and thus met criteria for PHTN. Of those children, only 146 (5.7%) had a corresponding *ICD-10* code (I10). Children who met criteria had greater odds of having a PHTN diagnosis code if they were older (119 of 1041 [11.4%] aged ≥12 years vs 7 of 493 [1.4%] aged ≤6 years); multiracial (4 of 37 [10.8%]) vs White (79 of 1563 [5.1%]) race; and have attended a clinic in rural (22 of 346 [6.4%]) vs urban (95 of 1714 [5.5%]) setting. There were no significant differences in diagnosis by sex, ethnicity, or BMI status.

The CDS tool was used to calculate systolic blood pressure percentile for 10 524 children (45.1%). Associations with CDS tool use are presented in the eResults and eTable 1 in Supplement 1. When the CDS tool was used, the odds of children having a guideline-adherent diagnosis code were significantly greater for children with blood pressure at or above the 90th percentile (*ICD-10* code R03.0: 4733 of 8436 [56.1%] vs 4077 of 14 892 [27.4%]) and blood pressure at or above the 95th percentile at 3 visits or more (*ICD-10* code I10: 118 of 1089 [10.8%] vs 28 of 1451 [1.9%]) (odds ratio, 2.14 [95% CI, 1.10-4.15]).

### Guideline-Adherent Management

Guideline-adherent management results were similar between blood pressure at or above the 90th and 95th percentiles; thus, results for blood pressure at or above the 95th percentile are presented in Table 3, the Figure, B, and in this section. Results for blood pressure at or above the 90th percentile are in eTable 2 in Supplement 1.

**Table 3.** Logistic Regression Associations With Receiving Guideline-Adherent Management Among Children With Elevated Blood Pressure Readings at or Above the 95th Percentile

Variable	Antihypertensive medication^a^			Lifestyle counseling^b^			Referral^c^		
	No. (%)	AOR (95% CI)	*P* value^d^	No. (%)	AOR (95% CI)	*P* value^d^	No. (%)	AOR (95% CI)	*P* value^d^
Age, y									
≤6	92 (1.7)	1.11 (1.10-1.13)	<.001	5195 (98.6)	0.95 (0.93-0.96)	<.001	418 (7.9)	0.97 (0.95-0.98)	<.001
7-11	432 (7.2)			5627 (93.8)			245 (4.1)		
≥12	307 (7.4)			4019 (96.8)			185 (4.5)		
Sex									
Boys	572 (6.7)	1 [Reference]		8161 (95.3)	1 [Reference]		447 (5.2)	1 [Reference]	
Girls	259 (3.8)	0.54 (0.46-0.63)	<.001	6680 (97.4)	1.87 (1.56-2.24)	<.001	401 (5.8)	1.08 (0.94-1.24)	.29
Race									
Asian	10 (2.0)	0.21 (0.11-0.39)	<.001	484 (99.0)	6.74 (2.74-16.62)	<.001	18 (3.7)	0.59 (0.35-0.98)	.04
Black	246 (6.2)	0.67 (0.55-0.83)	<.001	3776 (95.1)	1.36 (1.06-1.74)	.01	206 (5.2)	0.81 (0.63-1.03)	.09
Hispanic or Latino	18 (1.9)	0.59 (0.36-0.97)	.04	908 (98.3)	1.50 (0.90-2.52)	.12	47 (5.1)	0.74 (0.54-1.01)	.06
White	495 (5.5)	1 [Reference]		8718 (96.4)	1 [Reference]		550 (6.1)	1 [Reference]	
Multiracial	19 (8.7)	1.10 (0.66-1.81)	.72	207 (95.0)	1.09 (0.58-2.06)	.79	9 (4.1)	0.79 (0.39-1.59)	.51
Other	5 (2.5)	0.28 (0.11-0.70)	.006	192 (97.5)	2.20 (0.88-5.48)	.09	2 (1.0)	0.23 (0.06-0.96)	.04
Unknown	38 (6.5)	0.77 (0.52-1.13)	.18	556 (94.4)	1.19 (0.77-1.82)	.44	23 (3.9)	0.44 (0.25-0.77)	.004
Ethnicity									
Hispanic or Latino	236 (3.0)	0.31 (0.25-0.38)	<.001	6816 (95.0)	2.48 (1.94-3.16)	<.001	535 (6.8)	1.32 (1.05-1.66)	.02
Non-Hispanic or non-Latino	570 (7.9)	1 [Reference]		7638 (97.5)	1 [Reference]		229 (4.2)	1 [Reference]	
Unknown	25 (6.1)	0.80 (0.51-1.23)	.30	387 (94.4)	0.92 (0.58-1.46)	.72	14 (3.4)	0.89 (0.51-1.57)	.70
BMI status									
Underweight	13 (4.1)	0.84 (0.47-1.50)	.56	297 (94.0)	0.71 (0.44-1.15)	.16	21 (6.6)	0.75 (0.47-1.18)	.21
Normal	315 (5.2)	1 [Reference]		5731 (95.4)	1 [Reference]		535 (8.9)	1 [Reference]	
Overweight	115 (4.7)	0.82 (0.65-1.03)	.08	2346 (96.7)	1.44 (1.12-1.86)	.005	134 (5.5)	0.61 (0.50-0.74)	<.001
Obesity	388 (5.8)	0.90 (0.77-1.06)	.23	6467 (97.0)	1.73 (1.42-2.10)	<.001	168 (2.5)	0.28 (0.23-0.33)	<.001
Clinic setting									
Urban	490 (4.5)	1 [Reference]		10 500 (96.2)	1 [Reference]		732 (6.7)	1 [Reference]	
Suburban	120 (4.6)	1.01 (0.81-1.25)	.94	2552 (96.8)	1.02 (0.81-1.30)	.84	114 (4.3)	0.60 (0.49-0.74)	<.001
Rural	221 (11.8)	1.99 (1.62-2.45)	<.001	1789 (95.6)	1.30 (0.98-1.75)	.07	2 (0.1)	0.01 (0.00-0.06)	<.001

Abbreviations: AOR, adjusted odds ratio; BMI, body mass index.

^a^ Of 15 422 children and adolescents, 831 (5.4%) were prescribed antihypertensive medication.

^b^ Of 15 422 children and adolescents, 14 841 (96.2%) received lifestyle counseling.

^c^ Of 15 422 children and adolescents, 848 (5.5%) were referred to pediatric hypertension-related specialty services.

^d^ Compared with the reference category, values indicate the association with having guideline-adherent management documented in the electronic health record.

Antihypertensive medication was prescribed to 831 children (5.4%). Children prescribed antihypertensive medication were more likely to be older (307 of 4151 [7.4%] aged ≥12 years vs 92 of 5271 [1.7%] aged ≤6 years); boys (572 of 8563 [6.7%]) vs girls (259 of 6859 [3.8%]); White (495 of 9039 [5.5%]) vs Asian (10 of 489 [2.0%]), Black (246 of 3970 [6.2%]), Hispanic or Latino (18 of 924 [1.9%]), and other (5 of 197 [2.5%]) race; non-Hispanic or non-Latino (570 of 7176 [7.9%] vs Hispanic or Latino (236 of 7836 [3.0%]) ethnicity; and have attended a clinic in a rural (221 of 1871 [11.8%]) rather than an urban (490 of 10 915 [4.5%]) setting. There was no significant difference in prescriptions by BMI status.

Lifestyle counseling rates were high (14 841 [96.2%]). Children who received lifestyle counseling were more likely to be younger (5195 of 5271 [98.6%] aged ≤6 years vs 4019 of 4151 [96.8%] aged ≥12 years); girls (6680 of 6859 [97.4%]) vs boys (8161 of 8563 [95.3%]); Asian (484 of 489 [99.0%]) or Black (3776 of 3970 [95.1%]) vs White (8718 of 9039 [96.4%]) race; Hispanic or Latino (6816 of 7176 [95.0%]) vs non-Hispanic or non-Latino (7638 of 7836 [97.5%]) ethnicity; and classified with overweight (2346 of 2427 [96.7%]) or obesity (6467 of 6669 [97.0%]) vs normal (5731 of 6010 [95.4%]) BMI. There was no significant difference in counseling by clinic setting.

Referrals for specialty services related to elevated blood pressure were provided to 848 children (5.5%). Children who received a referral were more likely to be younger (418 of 5271 [7.9%] aged ≤6 years vs 185 of 4151 [4.5%] aged ≥12 years); White (550 of 9039 [6.1%]) vs Asian (18 of 489 [3.7%]), other (2 of 197 [1.0%]), or unknown (23 of 587 [3.9%]) race; Hispanic or Latino (535 of 7836 [6.8%]) vs non-Hispanic or non-Latino (229 of 7176 [3.2%]) ethnicity; classified with normal (535 of 7836 [6.8%]) vs overweight (134 of 2427 [5.5%]) or obesity (168 of 6660 [2.5%]) BMI; and have attended a clinic in an urban (732 of 10 915 [6.7%]) vs suburban (114 of 2636 [4.3%]) or rural (2 of 1871 [0.1%]) setting. There was no significant difference in referrals by sex.

### Guideline-Adherent Follow-up

Guideline-adherent follow-up results are presented in Table 4 and the Figure, C. Guideline-adherent follow-up was observed in 8651 of 19 049 children (45.4%) with blood pressure at or above the 90th percentile and 2598 of 15 164 (17.1%) with blood pressure at or above the 95th percentile. A total of 4197 children with blood pressure at the 90th to 94th percentiles were eligible for the follow-up visit analyses. Of those children, 2269 (54.1%) attended a follow-up visit within 6 months. Children who attended a guideline-adherent follow-up visit were more likely to be older (1559 of 2651 [58.8%] aged ≥12 years vs 189 of 462 [40.9%] aged ≤6 years); White (1415 of 2573 [55.0%]) vs Hispanic or Latino (102 of 232 [44.0%]) race; classified as non-Hispanic or non-Latino (978 of 1797 [54.2%]) vs unknown (55 or 119 [46.2%]) ethnicity; classified as underweight (27 of 41 [65.9%]) or obesity (1253 of 2150 [58.3%]) vs normal (651 of 1325 [49.1%]) BMI; and have attended a clinic in an urban (1775 of 3191 [55.6%]) vs suburban (305 of 586 [52.0%]) or rural (189 of 379 [49.9%]) setting. There were no significant differences by sex.

**Table 4.** Logistic Regression Associations With Attending a Guideline-Adherent Follow-up Visit Among Children With Elevated BP Readings

Variable	Attended a follow-up visit within 6 mo after BP reading between 90th and 94th percentile^a^			Attended a follow-up visit within 1 mo after BP reading ≥95th percentile^b^		
	No. (%)	AOR (95% CI)	*P* value^c^	No. (%)	AOR (95% CI)	*P* value^c^
Age, y						
≤6	189 (40.9)	1.07 (1.05-1.09)	<.001	544 (10.6)	1.10 (1.09-1.11)	<.001
7-11	521 (48.1)			953 (16.1)		
≥12	1559 (58.8)			1101 (26.7)		
Sex						
Boys	2521 (13.2)	1 [Reference]		1529 (18.1)	1 [Reference]	
Girls	1676 (8.8)	1.06 (0.94-1.21)	.35	1069 (15.9)	0.90 (0.82-0.98)	.02
Race						
Asian	44 (46.3)	0.70 (0.45-1.10)	.12	80 (16.7)	0.99 (0.76-1.29)	.93
Black	604 (54.6)	0.83 (0.67-1.02)	.08	611 (15.7)	0.79 (0.69-0.92)	.002
Hispanic or Latino	102 (44.0)	0.59 (0.45-0.78)	<.001	125 (13.7)	0.68 (0.55-0.83)	<.001
White	1415 (55.0)	1 [Reference]		1639 (18.4)	1 [Reference]	
Multiracial	23 (56.1)	1.20 (0.63-2.28)	.58	30 (14.3)	0.89 (0.60-1.33)	.58
Other	14 (48.3)	0.78 (0.37-1.64)	.51	24 (12.5)	0.69 (0.44-1.08)	.11
Unknown	67 (55.4)	1.06 (0.69-1.61)	.80	89 (15.5)	0.85 (0.65-1.11)	.23
Ethnicity						
Hispanic or Latino	1236 (54.2)	0.86 (0.71-1.05)	.14	1390 (18.0)	1.01 (0.89-1.15)	.89
Non-Hispanic or non-Latino	978 (54.4)	1 [Reference]		1144 (16.2)	1 [Reference]	
Unknown	55 (46.2)	0.66 (0.45-0.99)	.04	64 (15.9)	0.95 (0.71-1.27)	.75
BMI status						
Underweight	27 (65.9)	2.25 (1.15-4.37)	.02	41 (13.4)	0.97 (0.69-1.36)	.84
Normal	651 (49.1)	1 [Reference]		881 (14.9)	1 [Reference]	
Overweight	338 (49.6)	0.97 (0.80-1.17)	.72	374 (15.7)	0.93 (0.81-1.06)	.28
Obesity	1253 (58.3)	1.36 (1.18-1.57)	<.001	1302 (19.8)	1.09 (0.99-1.20)	.09
Clinic setting						
Urban	1775 (55.6)	1 [Reference]		1785 (16.6)	1 [Reference]	
Suburban	305 (52.0)	0.76 (0.64-0.91)	.003	428 (19.0)	0.92 (0.82-1.04)	.20
Rural	189 (49.9)	0.74 (0.57-0.94)	.02	295 (16.2)	0.91 (0.79-1.07)	.29

Abbreviations: AOR, adjusted odds ratio; BMI, body mass index; BP, blood pressure.

^a^ Of 4197 eligible children and adolescents, 2269 (54.1%) attended a follow-up visit.

^b^ Of 15 164 eligible children and adolescents, 2598 (17.1%) attended a follow-up visit.

^c^ Compared with the reference category, values indicate the association with having a guideline-adherent follow-up visit documented in the electronic health record.

Of 15 164 children with blood pressure at or above the 95th percentile who were eligible for the follow-up visit analyses, 2598 (17.1%) attended a follow-up visit within 1 month. Children who attended a guideline-adherent follow-up visit were more likely to be older (1101 of 4118 [26.7%] aged ≥12 years vs 544 of 5128 [10.6%] aged ≤6 years); boys (1529 of 8428 [18.1%]) vs girls (1069 of 6736 [15.9%]); and White (1639 of 8900 [18.4%]) vs Black (611 of 3896 [15.7%]) or Hispanic or Latino (125 of 911 [13.7%]) race. There were no significant differences by ethnicity, BMI status, or clinic setting.

## Discussion

With the publication of the 2017 CPG for the diagnosis and management of PHTN, the rate of children who meet diagnostic criteria for PHTN has increased.^5,7^ To our knowledge, this is the first analysis to examine the rates of guideline-adherent PHTN diagnosis and management documented in the EHR using the 2017 CPG. Compared with a prior study in this population using the Fourth Report,^12^ guideline-adherent diagnosis rates for PHTN using the 2017 CPG were considerably lower (5.7% vs 42%) while the rates of children diagnosed with prehypertension or elevated blood pressure without a diagnosis of hypertension were higher (37.8% vs 17%). Consistent with prior studies, children who were boys, older (aged ≥13 years), and classified with overweight or obesity were more likely to be diagnosed with PHTN or elevated blood pressure.^1,9,27^ Additionally, children seen in urban-based clinics were more likely to have a guideline-adherent diagnosis code for elevated blood pressure, but not PHTN.

Behavioral change is the first-line intervention recommended for treating elevated blood pressure among children.^4^ Accordingly, 96.2% of patients in this sample received some form of lifestyle counseling, suggesting that recording a guideline-adherent diagnosis had a minimal effect on delivery of guideline-adherent counseling. Children classified with overweight and obesity had 33% and 56% greater odds, respectively, of receiving lifestyle counseling. Among children meeting criteria for elevated blood pressure or PHTN, 5.4% were prescribed antihypertensive medication and 5.5% received a referral for potential blood pressure–related specialty care. Interestingly, antihypertensive medications were more likely to be prescribed to children attending a clinic in a suburban or rural setting, while referrals were more likely to be given to those in in an urban setting. A recent survey of pediatric clinicians in the AllianceChicago network^28^ found that 71% of clinicians agreed that selecting and prescribing antihypertensive medications was a barrier to PHTN management, and 74% preferred to refer children with PHTN to a specialist for treatment. These findings highlight the need to ensure that pediatric clinicians, particularly those in rural settings where specialty services may not be as readily available, have adequate comfort and familiarity with antihypertensive medications to manage PHTN per the 2017 CPG. Furthermore, reducing barriers to accessing specialty care (eg, telehealth) is critical for blood pressure management in children.

Per the 2017 CPG,^4^ children with blood pressure at or above the 90th and 95th percentiles should attend a follow-up visit within and 6 months and 2 weeks, respectively, for a follow-up blood pressure reading. In the present sample, 54.1% of children with blood pressure at or above the 90th percentile attended a 6-month follow-up visit but only 17.1% of children with blood pressure at or above the 95th percentile attended a follow-up visit within 1 month, similar to a prior study (21%).^25^ Lack of patient follow-up inherently limits the ability of clinicians to confirm 3 occurrences of blood pressure at or above the 95th percentile and confidently diagnose and treat PHTN. Specific strategies targeting patient attendance, using home or ambulatory blood pressure monitoring,^29^ and reducing barriers to accessing specialty care might improve follow-up and increase rates of guideline-adherent PHTN diagnosis and subsequent management.

We observed several differences in guideline adherence by patient-level characteristics. For example, while guideline-adherent elevated blood pressure diagnosis was highest among Black children, rates of antihypertensive medication prescription, referral, and follow-up were higher among White children. Similarly, a higher proportion of boys than girls was prescribed antihypertensive medication and attended follow-up visits. These substantial racial and sex differences in PHTN management may be suggestive of implicit bias.^21^ Using EHR-based tools, such as the CDS tool, can be used to mitigate the harmful effects of implicit racial and sex bias and increase equitable identification and management of PHTN.^30^

The findings from this study underscore the evidence-to-practice gap in guideline implementation.^10,11^ Clinical decision support tools are often implemented to support clinicians and improve clinical care, such as increasing adherence to clinical guidelines. Unfortunately, the PHTN CDS tool implemented in AllianceChicago clinics was used on fewer than half the patients with elevated blood pressure readings (≥90th percentile), and uptake was significantly lower in rural compared with urban clinics. These findings are consistent with prior studies in PHTN and more broadly, indicating that these tools are effective but have low adoption rates.^23,31^ Thus, comprehensive implementation strategies are needed to increase uptake of effective CDS tools to promote guideline-adherent PHTN diagnosis and management. For example, a stakeholder advisory panel consisting of AllianceChicago pediatricians and academic partners identified 18 discrete implementation strategies needed to improve CPG adherence, including education and training, workflow changes, and leadership support.^19^

### Limitations

This study has some limitations. First, the focus on high-risk children with at least 1 blood pressure reading at or above the 90th percentile limits the generalizability of these findings. Second, data extracted from the EHR limits the information we have, including whether the management options (medications, counseling, and referrals) were specific to elevated blood pressure (eg, lifestyle counseling focused on weight management); the method of blood pressure measurement (eg, manual vs automatic, cuff size) is unknown; follow-up blood pressure measures may have been normal but not entered in the EHR; or diagnoses entered as text in notes may not be included as *ICD-10* codes. Finally, the true prevalence of PHTN is unknown, as the diagnosis requires 3 instances of elevated blood pressure at or above the 95th percentile, and the rates of follow-up visit attendance were low.

## Conclusions

Adhering to the 2017 CPG is critical for reducing PHTN-related morbidity and mortality among children, adolescents, and adults.^8,13,14,16,32^ However, in this cross-sectional study, elevated blood pressure and PHTN were underdiagnosed and undertreated among children seen in safety-net clinics, and a CDS tool to facilitate diagnosis was underused. Comprehensive implementation strategies are needed to address this evidence-to-practice gap, including strategies to support the implementation of health information technology tools.

## Notes

Supplement 1. **eMethods.** Guideline-Adherent Management and CDS Tool **eResults.** CDS Tool Use **eTable 1.** Logistic Regression Associations With Having Clinical Decision Support (CDS) Tool–Calculated BP Among Children With BP ≥90th Percentile (n = 23 334) **eTable 2.** Logistic Regression Associations With Receiving Guideline-Adherent Management Among Children With Elevated BP ≥90th Percentile (n = 23 334) **eFigure 1.** AllianceChicago Network Locations **eFigure 2.** Clinical Decision Support (CDS) Tool Supporting the 2017 Clinical Practice Guideline for the Diagnosis and Management of Pediatric Hypertension

Supplement 2.
Data Sharing Statement

## References

[1] Bell CS, Samuel JP, Samuels JA. Prevalence of hypertension in children. Hypertension. 2019;73(1):148-152. doi:10.1161/HYPERTENSIONAHA.118.11673 30571555 PMC6291260

[2] Song P, Zhang Y, Yu J, . Global prevalence of hypertension in children: a systematic review and meta-analysis. JAMA Pediatr. 2019;173(12):1154-1163. doi:10.1001/jamapediatrics.2019.3310 31589252 PMC6784751

[3] Skinner AC, Ravanbakht SN, Skelton JA, Perrin EM, Armstrong SC. Prevalence of obesity and severe obesity in US children, 1999-2016. Pediatrics. 2018;141(3):141. doi:10.1542/peds.2017-3459 29483202 PMC6109602

[4] Flynn JT, Kaelber DC, Baker-Smith CM, ; Subcommittee on Screening and Management of High Blood Pressure in Children. Clinical practice guideline for screening and management of high blood pressure in children and adolescents. Pediatrics. 2017;140(3):e20171904. doi:10.1542/peds.2017-1904 28827377

[5] Yang L, Kelishadi R, Hong YM, . Impact of the 2017 American Academy of Pediatrics Guideline on Hypertension Prevalence compared with the Fourth Report in an international cohort. Hypertension. 2019;74(6):1343-1348. doi:10.1161/HYPERTENSIONAHA.119.13807 31630571

[6] National High Blood Pressure Education Program Working Group on High Blood Pressure in Children and Adolescents. The Fourth Report on the Diagnosis, Evaluation, and Treatment of High Blood Pressure in children and adolescents. Pediatrics. 2004;114(suppl 2, 4th Report):555-576. doi:10.1542/peds.114.S2.555 15286277

[7] Al Kibria GM, Swasey K, Sharmeen A, Day B. Estimated change in prevalence and trends of childhood blood pressure levels in the United States after application of the 2017 AAP guideline. Prev Chronic Dis. 2019;16:E12. doi:10.5888/pcd16.180528 30702999 PMC6362707

[8] Khoury M, Khoury P, Bazzano L, . Prevalence implications of the 2017 American Academy of Pediatrics hypertension guideline and associations with adult hypertension. J Pediatr. 2022;241:22-28.e4. doi:10.1016/j.jpeds.2021.09.056 34619113 PMC8924915

[9] Hansen ML, Gunn PW, Kaelber DC. Underdiagnosis of hypertension in children and adolescents. JAMA. 2007;298(8):874-879. doi:10.1001/jama.298.8.874 17712071

[10] Patel ND, Newburn A, Brier ME, Chand DH. Pediatric hypertension: are pediatricians following guidelines? J Clin Hypertens (Greenwich). 2016;18(12):1230-1234. doi:10.1111/jch.12915 27659546 PMC8031817

[11] Rao G, Naureckas S, Datta A, . Pediatric hypertension: diagnostic patterns derived from electronic health records. Diagnosis (Berl). 2018;5(3):157-160. doi:10.1515/dx-2018-0010 30130249

[12] Moin A, Mohanty N, Tedla YG, . Under-recognition of pediatric hypertension diagnosis: examination of 1 year of visits to community health centers. J Clin Hypertens (Greenwich). 2021;23(2):257-264. doi:10.1111/jch.14148 33373088 PMC8030016

[13] Khoury M, Urbina EM. Cardiac and vascular target organ damage in pediatric hypertension. Front Pediatr. 2018;6:148. doi:10.3389/fped.2018.00148 29881718 PMC5976785

[14] Urbina EM, Mendizábal B, Becker RC, . Association of blood pressure level with left ventricular mass in adolescents. Hypertension. 2019;74(3):590-596. doi:10.1161/HYPERTENSIONAHA.119.13027 31327264

[15] Dawson AE, Kallash M, Spencer JD, Wilson CS. The pressure’s on: understanding neurocognitive and psychological associations with pediatric hypertension to inform comprehensive care. Pediatr Nephrol. 2021;36(12):3869-3883. doi:10.1007/s00467-021-05077-w 33890179

[16] Du T, Fernandez C, Barshop R, Chen W, Urbina EM, Bazzano LA. 2017 Pediatric hypertension guidelines improve prediction of adult cardiovascular outcomes. Hypertension. 2019;73(6):1217-1223. doi:10.1161/HYPERTENSIONAHA.118.12469 31006329 PMC6673663

[17] Theodore RF, Broadbent J, Nagin D, . Childhood to early-midlife systolic blood pressure trajectories: early-life predictors, effect modifiers, and adult cardiovascular outcomes. Hypertension. 2015;66(6):1108-1115. doi:10.1161/HYPERTENSIONAHA.115.05831 26558818 PMC4646716

[18] Bello JK, Mohanty N, Bauer V, Rittner SS, Rao G. Pediatric hypertension: provider perspectives. Glob Pediatr Health. 2017;4(4):2333794X1771263. doi:10.1177/2333794X17712637 28620629 PMC5464512

[19] Knapp AA, Carroll AJ, Mohanty N, . A stakeholder-driven method for selecting implementation strategies: a case example of pediatric hypertension clinical practice guideline implementation. Implement Sci Commun. 2022;3(1):25. doi:10.1186/s43058-022-00276-4 35256017 PMC8900435

[20] Marewski JN, Gigerenzer G. Heuristic decision making in medicine. Dialogues Clin Neurosci. 2012;14(1):77-89. doi:10.31887/DCNS.2012.14.1/jmarewski 22577307 PMC3341653

[21] Sabin JA, Greenwald AG. The influence of implicit bias on treatment recommendations for 4 common pediatric conditions: pain, urinary tract infection, attention deficit hyperactivity disorder, and asthma. Am J Public Health. 2012;102(5):988-995. doi:10.2105/AJPH.2011.300621 22420817 PMC3483921

[22] Meisner JK, Yu S, Lowery R, Liang W, Schumacher KR, Burrows HL. Clinical decision support tool for elevated pediatric blood pressures. Clin Pediatr (Phila). 2022;61(5-6):428-439. doi:10.1177/00099228221087804 35383471

[23] Vuppala S, Turer CB. Clinical decision support for the diagnosis and management of adult and pediatric hypertension. Curr Hypertens Rep. 2020;22(9):67. doi:10.1007/s11906-020-01083-9 32852616 PMC7450038

[24] Health Resources & Services Administration. National Health Center Program Uniform Data System (UDS) Awardee Data: UDS Data Five-Year Summary. Health Center Program UDS Data; 2022.

[25] Daley MF, Sinaiko AR, Reifler LM, . Patterns of care and persistence after incident elevated blood pressure. Pediatrics. 2013;132(2):e349-e355. doi:10.1542/peds.2012-2437 23821694 PMC3727670

[26] Stata Statistical Software. Release 17. StataCorp LLC; 2021.

[27] Rerksuppaphol L, Rerksuppaphol S. Prevalence and risk factors of hypertension in schoolchildren from central Thailand: a cross-sectional study. Int J Prev Med. 2021;12:28. 34249277 10.4103/ijpvm.IJPVM_110_20 PMC8218803

[28] Carroll AJ, Tedla YG, Padilla R, . Correlates of adherence to the 2017 Clinical Practice Guidelines for pediatric hypertension in safety-net clinics: a two-year cross-sectional study. medRxiv. Preprint posted online October 6, 2022. doi:10.1101/2022.10.03.22280528 PMC10105315 37058305

[29] Peterson CG, Miyashita Y. The use of ambulatory blood pressure monitoring as standard of care in pediatrics. Front Pediatr. 2017;5:153. doi:10.3389/fped.2017.00153 28713799 PMC5492637

[30] Rumball-Smith J, Bates DW. The electronic health record and health IT to decrease racial/ethnic disparities in care. J Health Care Poor Underserved. 2018;29(1):58-62. doi:10.1353/hpu.2018.0006 29503287

[31] Kouri A, Yamada J, Lam Shin Cheung J, Van de Velde S, Gupta S. Do providers use computerized clinical decision support systems? a systematic review and meta-regression of clinical decision support uptake. Implement Sci. 2022;17(1):21. doi:10.1186/s13012-022-01199-3 35272667 PMC8908582

[32] Urbina EM, Khoury PR, McCoy C, Daniels SR, Kimball TR, Dolan LM. Cardiac and vascular consequences of pre-hypertension in youth. J Clin Hypertens (Greenwich). 2011;13(5):332-342. doi:10.1111/j.1751-7176.2011.00471.x 21545394 PMC3092159

