# Supplementary material for: Adherence to the 2017 Clinical Practice Guidelines for Pediatric Hypertension in Safety-Net Clinics
Source: JAMA Netw Open. 2023 Apr 14;6(4):e237043. doi: 10.1001/jamanetworkopen.2023.7043 (PMC10105315; doi:10.1001/jamanetworkopen.2023.7043)
Supplement: Supplement 1. — eMethods. Guideline-Adherent Management and CDS Tool eResults. CDS Tool Use eTable 1. Logistic Regression Associations With Having Clinical Decision Support (CDS) Tool–Calculated BP Among Children With BP ≥90th Percentile (n = 23 334) eTable 2. Logistic Regression Associations With Receiving Guideline-Adherent Management Among Children With Elevated BP ≥90th Percentile (n = 23 334) eFigure 1. AllianceChicago Network Locations eFigure 2. Clinical Decision Support (CDS) Tool Supporting the 2017 Clinical Practice Guideline for the Diagnosis and Management of Pediatric Hypertension [file jamanetwopen-e237043-s001.pdf]

Supplementary Online Content

Carroll AJ, Tedla YG, Padilla R, et al. Adherence to the 2017 Clinical Practice Guidelines for pediatric hypertension in safety-net clinics. *JAMA Netw Open*. 2023;6(4):e237043. doi:10.1001/jamanetworkopen.2023.7043

eMethods. Guideline-Adherent Management and CDS Tool

eResults. CDS Tool Use

eTable 1. Logistic Regression Associations With Having Clinical Decision Support (CDS) Tool–Calculated BP Among Children With BP ≥90th Percentile (n = 23 334)

eTable 2. Logistic Regression Associations With Receiving Guideline-Adherent Management Among Children With Elevated BP ≥90th Percentile (n = 23 334)

eFigure 1. AllianceChicago Network Locations

eFigure 2. Clinical Decision Support (CDS) Tool Supporting the 2017 Clinical Practice Guideline for the Diagnosis and Management of Pediatric Hypertension

This supplementary material has been provided by the authors to give readers additional information about their work.

© 2023 Carroll AJ et al. *JAMA Network Open*.

eMethods. Guideline-Adherent Management and CDS Tool

Guideline-adherent management

Antihypertensive medications extracted from the AllianceChicago EHR included Ace Inhibitors (Lisinopril, Enalapril, Captopril), Ace receptor blockers (Losartan, Valsartan), Calcium channel blockers (Amlodipine, Isradipine, Nifedipine), Beta blockers (Propranolol, Metoprolol, Labetalol, Carvedilol, Atenolol), Alpha Blockers (Doxazosin), Alpha 2 agonist (Clonidine), Diuretics, (Furosemide, Chlorothiazide, Metolazone, Spironolactone, Amiloride), and Vasodilator.

CDS tool

The CDS tool was made available across the AllianceChicago network starting 1/1/2018 to prompt adherence to the newly-released 2017 CPG for identifying and managing pHTN. The CDS tool included a BP interpretation form in well-child encounters, color indicators on vitals form for abnormal blood pressure, prompts for re-assessing bp when abnormal, tools to average repeat measurements, and opportunities to add appropriate ICD codes. Extensive training was provided around the 2017 CPG and the introduction of the CDS prior to the CDS implementation, including presentations, resources, reminders, and discussions. Screenshots of the CDS tool which were used during the training are provided in Supplemental eFigure 2.

eResults. CDS Tool Use

Of the full sample (N=23,334), the CDS tool was used to calculate the systolic BP percentile for 10,524 (45.1%) children. The CDS tool was more likely to be used for children who were girls (OR: 1.139, 95%CI: 1.075, 1.205), younger (OR: 0.809, 95%CI: 0.803, 0.815), Hispanic/Latino (OR: 1.204, 95%CI: 1.103, 1.314) or unknown ethnicity (OR: 1.222, 95%CI: 1.021, 1.462), and seen in an urban setting compared to those seen in a rural (OR: 0.603, 95%CI: 0.544, 0.668) or unknown clinic setting (OR: 0.542, 95%CI: 0.429, 0.685). There was no significant difference in the use of the CDS tool by BMI status.

© 2023 Carroll AJ et al. *JAMA Network Open*.

eTable 1. Logistic Regression Associations With Having Clinical Decision Support (CDS) Tool–Calculated BP Among Children With BP ≥90th Percentile (n = 23 334)

	Systolic blood pressure		Diastolic blood pressure	
Variable	n (%)	AOR (95% CI)	n (%)	AOR (95% CI)
Age, y	---	0.81 (0.80-0.82) ***	---	0.81 (0.80-0.82) ***
≤6	5150 (63.3%)	---	5152 (63.3%)	---
7-11	4498 (48.1%)	---	4500 (48.1%)	---
≥12	876 (15.0%)	---	876 (15.0%)	---
Sex				
Boys	5462 (42.6%)	Ref	5464 (42.7%)	Ref
Girls	5062 (48.1%)	1.14 (1.07-1.20) ***	5064 (48.1%)	1.14 (1.07-1.20) ***
Race				
Asian	342 (47.8%)	1.07 (0.89-1.27)	342 (47.8%)	1.07 (0.89-1.27)
Black	2708 (44.5%)	1.07 (0.98-1.18)	2710 (44.5%)	1.07 (0.98-1.18)
Hispanic or Latino	522 (36.8%)	0.59 (0.52-0.67) ***	522 (36.8%)	0.59 (0.52-0.66) ***
White	6264 (45.8%)	Ref	6266 (45.9%)	Ref
Multiracial	128 (40.5%)	0.66 (0.52-0.85) **	128 (40.5%)	0.66 (0.51-0.85) **
Other	131 (46.1%)	0.98 (0.75-1.27)	131 (46.1%)	0.98 (0.75-1.27)
Unknown	429 (50.2%)	1.14 (0.96-1.35)	429 (50.2%)	1.14 (0.96-1.35)
Ethnicity				
Hispanic or Latino	5563 (46.4%)	1.22 (1.12-1.34) ***	5564 (46.5%)	1.22 (1.12-1.34) ***
Non-Hispanic or non-Latino	4636 (43.4%)	Ref	4639 (43.4%)	Ref
Unknown	325 (48.6%)	1.23 (1.03-1.47) *	325 (48.6%)	1.23 (1.03-1.47) *
BMI status				
Underweight	272 (56.0%)	1.06 (0.87-1.29)	272 (56.0%)	1.06 (0.87-1.29)
Normal	4913 (50.5%)	Ref	4916 (50.5%)	Ref
Overweight	1689 (44.4%)	0.95 (0.87-1.03)	1690 (44.5%)	0.95 (0.87-1.03)
Obesity	3650 (39.2%)	1.01 (0.95-1.08)	3650 (39.2%)	1.01 (0.95-1.08)
Clinic setting				
Urban	7748 (46.0%)	Ref	7751 (46.0%)	Ref
Suburban	1629 (48.7%)	1.03 (0.95-1.11)	1629 (48.7%)	1.03 (0.95-1.11)
Rural	966 (36.1%)	0.61 (0.55-0.67) ***	967 (36.2%)	0.61 (0.55-0.67) ***

© 2023 Carroll AJ et al. *JAMA Network Open*.

* $p<.05$, * *$p<.01$, and *** $p<.001$ indicates a significant association with a clinician using the CDS tool to calculate blood pressure percentile in the EHR.
Abbreviations: AOR: adjusted odds ratio. BP: blood pressure. CI: confidence interval. BMI: body mass index. CDS: clinical decision support. EHR: electronic health record. Ref: reference category.

© 2023 Carroll AJ et al. *JAMA Network Open*.

eTable 2. Logistic Regression Associations With Receiving Guideline-Adherent Management Among Children With Elevated BP ≥90th Percentile (n = 23 334)

	Anti-hypertensive medication		Lifestyle counseling		Referral	
Variable	N (%)	AOR (95% CI)	N (%)	AOR (95% CI)	N (%)	AOR (95% CI)
Age, y	---	1.12 (1.10 - 1.13) ***	---	0.94 (0.93 - 0.96) ***	---	0.96 (0.95 - 0.98) ***
≤6	111 (1.4%)	---	8030 (98.7%)	---	633 (7.8%)	---
7-11	509 (5.4%)	---	8876 (94.9%)	---	394 (4.2%)	---
≥12	338 (5.8%)	---	5659 (96.9%)	---	262 (4.5%)	---
Sex						
Boys	649 (5.1%)	Ref	12291 (95.9%)	Ref	678 (5.3%)	Ref
Girls	309 (2.9%)	0.57 (0.50 - 0.66) ***	10274 (97.6%)	1.75 (1.50 - 2.04) ***	611 (5.8%)	1.05 (0.69 - 1.05)
Race						
Asian	10 (1.4%)	0.19 (0.10 – 0.37) ***	706 (98.7%)	4.64 (2.36 – 9.17) ***	31 (4.3%)	0.76 (0.51 – 1.14) *
Black	293 (4.8%)	0.71 (0.59 – 0.86) **	5828 (95.8%)	1.32 (1.07 - 1.64) *	316 (5.2%)	0.85 (0.69 - 1.04)
Hispanic or Latino	22 (1.6%)	0.62 (0.40 – 0.97) *	1389 (98.0%)	1.18 (0.79 – 1.75)	72 (5.1%)	0.71 (0.55 – 0.91) **
White	563 (4.1%)	Ref	13244 (96.9%)	Ref	830 (6.1%)	Ref
Multiracial	21 (6.6%)	1.14 (0.71 – 1.82)	302 (95.6%)	1.03 (0.59 – 1.79)	14 (4.4%)	0.92 (0.53 – 1.62)
Other	6 (2.1%)	0.32 (0.14 – 0.73) **	279 (98.2%)	2.70 (1.90 – 6.64) *	3 (1.1%)	0.26 (0.08 – 0.83) *
Unknown	43 (5.0%	0.85 (0.60 - 1.23)	817 (95.6%)	1.14 (0.78 - 1.67)	23 (2.7%)	0.46 (0.29 - 0.72) **
Ethnicity						
Hispanic or Latino	279 (2.3%)	0.33 (0.27 - 0.40) ***	11701 (97.7%)	2.35 (1.90 – 3.90) ***	816 (6.8%)	1.38 (1.14 - 1.66) **

© 2023 Carroll AJ et al. *JAMA Network Open*.

Non-Hispanic or non-Latino	650 (6.1%)	Ref	10224 (95.7%)	Ref	451 (4.2%)	Ref
Unknown	29 (4.3%)	0.73 (0.49 - 1.09)	640 (95.7%)	1.04 (0.69 - 1.55)	22 (3.3%)	0.86 (0.55 - 1.36)
BMI status						
Underweight	15 (3.1%)	0.88 (0.52 - 1.50)	459 (94.4%)	0.64 (0.42 – 0.95) *	36 (7.4%)	0.92 (0.65 - 1.31)
Normal	371 (3.8%)	Ref	9356 (96.2%)	Ref	811 (8.3%)	Ref
Overweight	141 (3.7%)	0.91 (0.74 - 1.11)	3687 (97.0%)	1.33 (1.07 - 1.65) *	219 (5.8%)	0.67 (0.57 - 0.78) ***
Obesity	431 (4.6%)	1.00 (0.86 - 1.16)	9063 (98.2%)	1.56 (1.32 – 1.85) ***	223 (2.4%)	0.28 (0.24 - 0.32) ***
Clinic setting						
Urban	579 (3.4%)	Ref	16309 (96.7%)	Ref	1136 (6.7%)	Ref
Suburban	128 (3.8%)	1.02 (0.83 - 1.25)	3684 (96.9%)	0.95 (0.77 - 1.17)	151 (4.0%)	0.55 (0.46 - 0.66) ***
Rural	251 (9.4%)	2.14 (1.77 - 2.59) ***	2572 (96.2%)	1.28 (0.99 - 1.65)	2 (0.1%)	0.01 (0.00 - 0.04) ***

* *p*<.05, * **p*<.01, and *** *p*<.001 indicates a significant association with receiving guideline-adherent management documented in the EHR. Abbreviations: BP: blood pressure. EHR: electronic health record. pHTN: pediatric hypertension. AOR: adjusted odds ratio. CI: confidence interval. BMI: body mass index. Ref: reference category.

© 2023 Carroll AJ et al. *JAMA Network Open*.

eFigure 1. AllianceChicago Network Locations

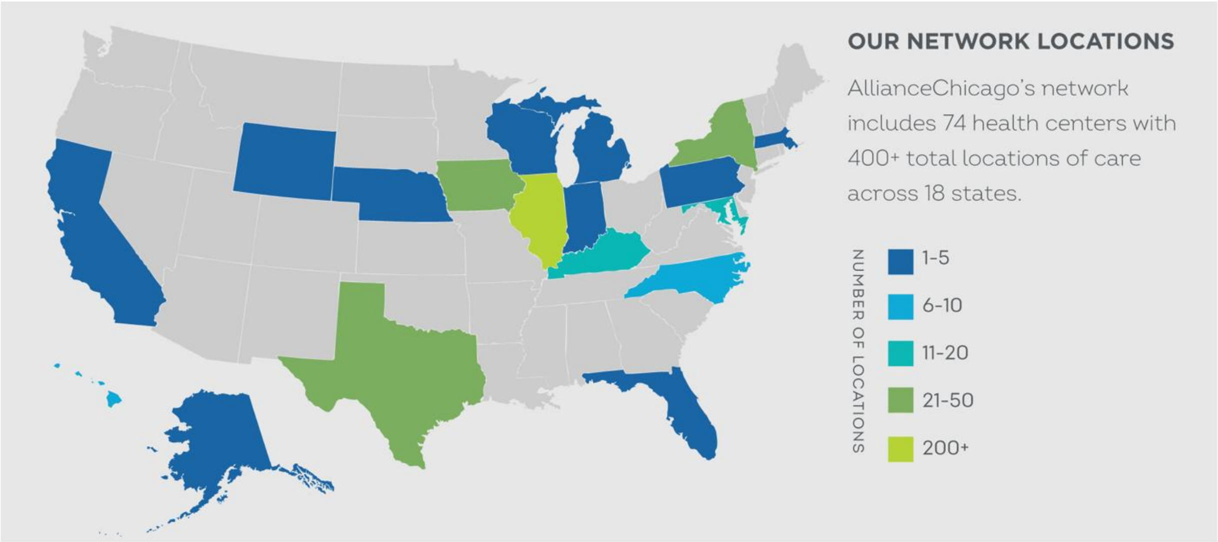

© 2023 Carroll AJ et al. *JAMA Network Open*.

eFigure 2. Clinical Decision Support (CDS) Tool Supporting the 2017 Clinical Practice Guideline for the Diagnosis and Management of Pediatric Hypertension

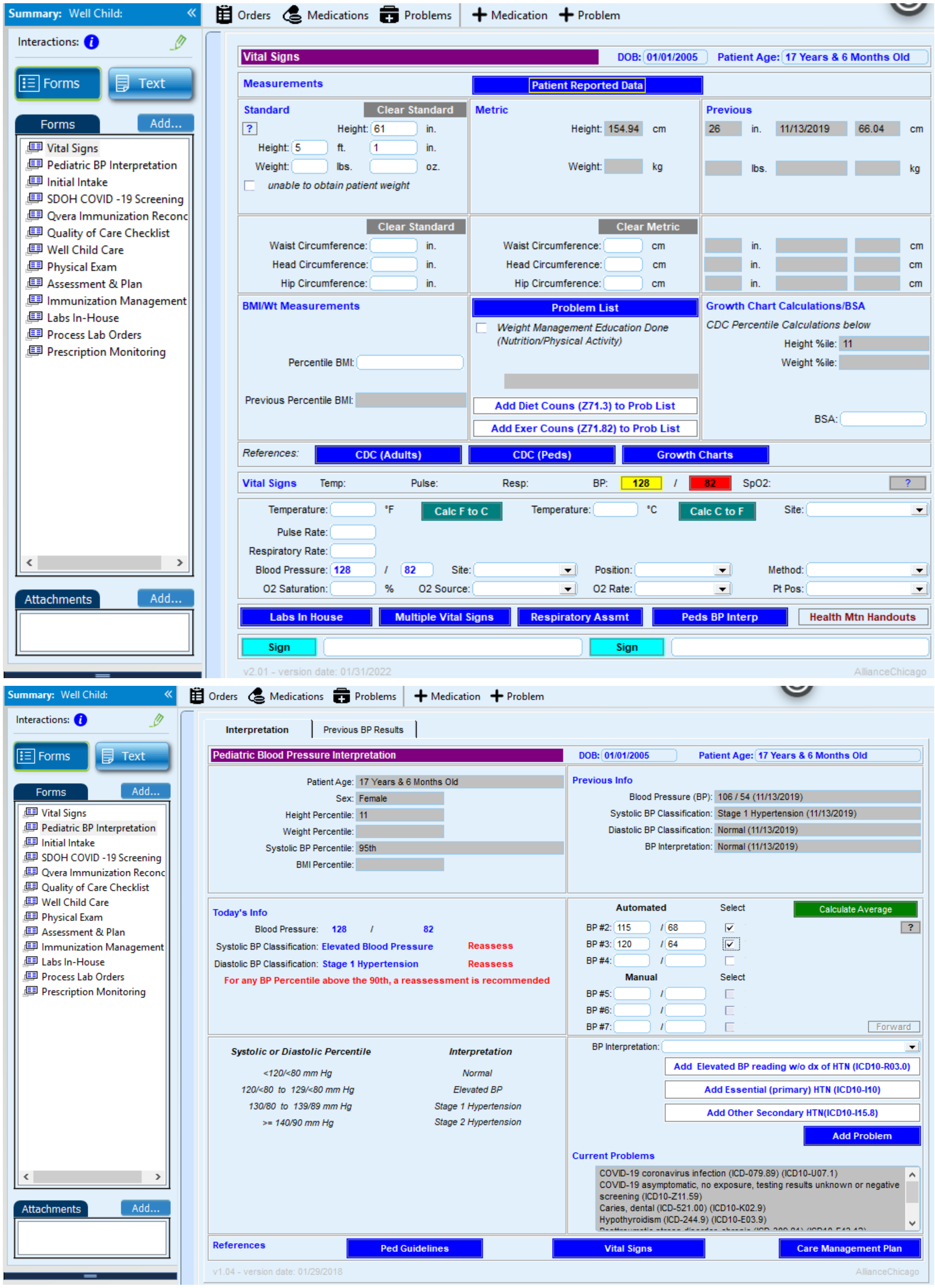

© 2023 Carroll AJ et al. *JAMA Network Open*.

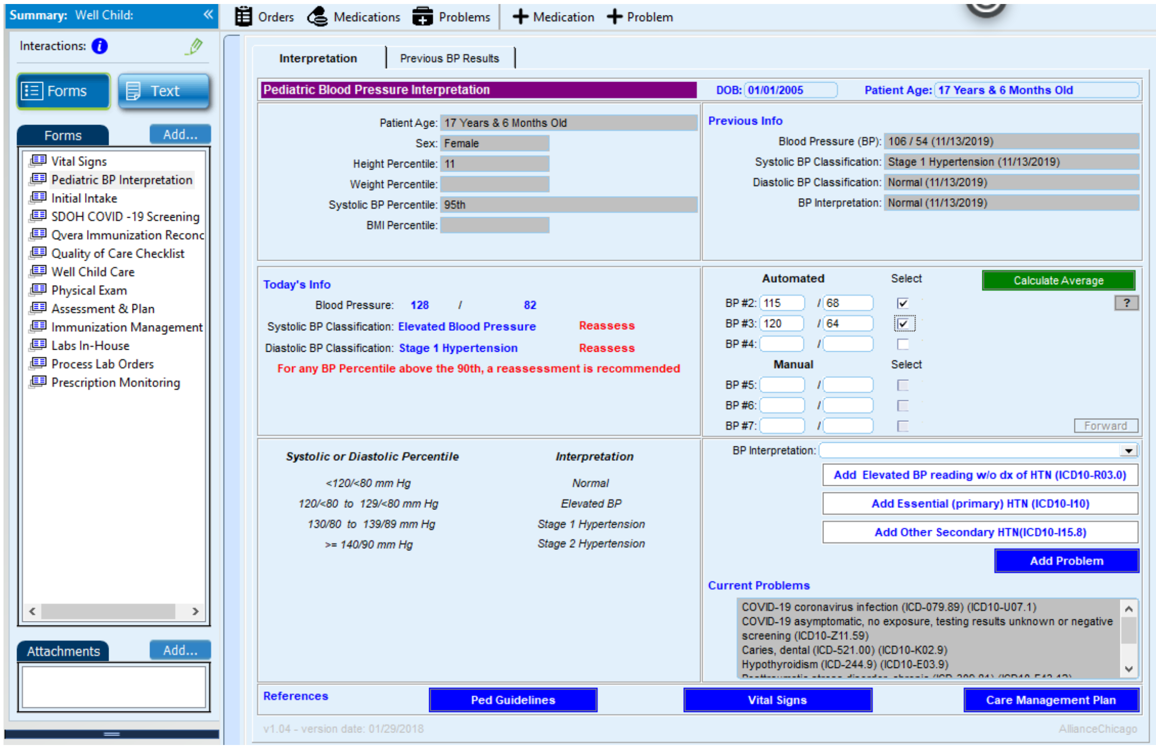

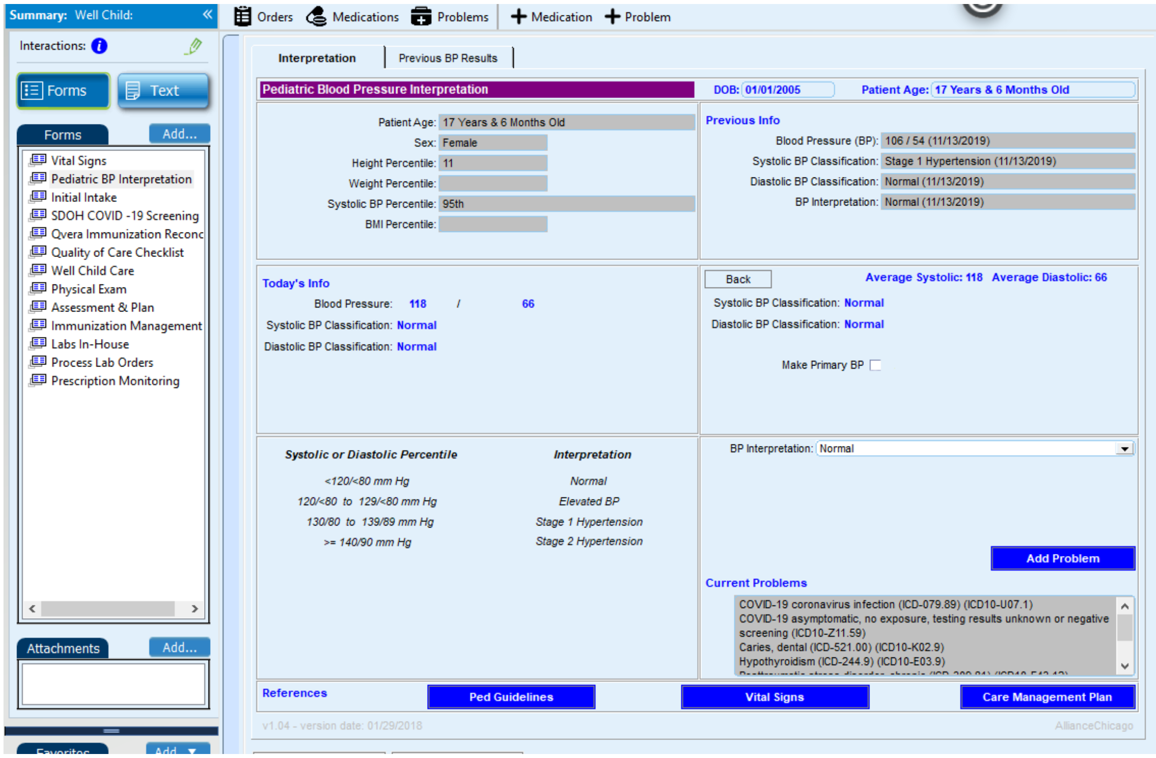

© 2023 Carroll AJ et al. *JAMA Network Open*.
