# Supplementary material for: Adherence to the 2017 Clinical Practice Guidelines for Pediatric Hypertension in Safety-Net Clinics
Source: JAMA Netw Open. 2023 Apr 14;6(4):e237043. doi: 10.1001/jamanetworkopen.2023.7043 (PMC10105315; doi:10.1001/jamanetworkopen.2023.7043)
Supplement: Supplement 2. — Data Sharing Statement [file jamanetwopen-e237043-s002.pdf]

Data Sharing Statement

Carroll. Adherence to the 2017 Clinical Practice Guidelines for Pediatric Hypertension in Safety-Net Clinics. *JAMA Netw Open*. Published April 14, 2023. doi:10.1001/jamanetworkopen.2023.7043

Data
Data available: No

Additional Information
Explanation for why data not available: As the data are part of the Enterprise Data Warehouse for AllianceChicago, we are unable to provide the dataset to other researchers beyond our team. However, reasonable requests from others may be considered on a case by case basis.
